# TRPC6 in Human Peripheral Nerves—An Investigation Using Immunohistochemistry

**DOI:** 10.3390/neurosci6020044

**Published:** 2025-05-19

**Authors:** Cedric Raming, Carola Meier, Thomas Tschernig

**Affiliations:** Institute of Anatomy and Cell Biology, Saarland University, Medical Campus, 66424 Homburg, Germany; ced.raming@gmail.com (C.R.); carola.meier@uks.eu (C.M.)

**Keywords:** TRPC6, cation channel, human nerves, peripheral nerves, immunohistochemistry

## Abstract

Since its discovery, TRPC6 has been associated with a variety of physiological and pathophysiological processes in different tissues. It functions as a non-selective cation channel and belongs to the group of TRP channels. Its importance in the development of pain hypersensitivity is becoming increasingly apparent. This condition has already been associated with increased expression of TRPC6 in dorsal root ganglia. Apart from the fact that most of the evidence has been obtained from samples of animal origin, it remains unclear whether the channel is also expressed in peripheral nerves outside the dorsal root ganglia. The aim of this work was therefore to examine peripheral nerves from human samples for TRPC6. For this purpose, samples of both the sciatic and ulnar nerves were taken from a total of eight body donors and analyzed by immunohistochemistry. Both longitudinal and transverse sections were obtained from the samples and stained. In total, 43 of 48 histological sections showed a positive immunosignal. There were no major differences between the sciatic and ulnar nerves with regard to staining. There was a slight difference in the staining intensity of transverse and longitudinal sections. The longitudinal sections of both nerves were consistently colored slightly more intensely. However, the inter-individual differences between the donors were more pronounced. Interestingly, the samples of a donor who suffered from chronic pain syndrome during his lifetime were particularly strongly stained. This is consistent with the knowledge gained to date, largely from animal experiments, that the channel shows increased expression in pain conditions in dorsal root ganglia. In the future, TRPC6 could therefore be a target in pain therapy.

## 1. Introduction

The TRPC6 channel belongs to the group of TRPC channels, which in turn can be subordinated to the TRP channel superfamily [1]. The TRP superfamily contains a total of seven subgroups of channels [1]. The affiliation of a channel to the TRP superfamily is primarily based on sequence homology [2]. The TRP channels share a number of functional and structural similarities. They are permeable to cations and have a similar structure [2]. The subgroup of TRPC channels was the first group of TRP channels to be discovered [3]. There are seven TRPC channels in total, some of which can be divided into subgroups based on their amino acid sequence as follows: TRPC1, TRPC2, TRPC3/6/7, TRPC4/5 [1].

TRPC6 was first described in 1997 [4]. The channel is non-selective for cations, whereby the permeability for calcium ions is somewhat higher than for other cations [5]. The channel forms both homotetramers and heterotetramers [5]. TRPC3 and TRPC7 in particular serve as partners for tetramer formation [5]. However, TRPC6 can also form heterotetramers with TRPC1, TRPC4, and TRPC5 [6]. TRPC6 plays a role in numerous physiological and pathophysiological processes [7]. Nevertheless, research, which is mainly carried out using gene knockout and small-molecule modulators, is difficult [7]. This is mainly due to the spread of the channel in the body and the resulting difficulty in specifying certain functions [7]. In addition, the interpretation of study results is complicated due to the pronounced heteromer formation of TRPC channels and the associated change in function [7].

The channel is expressed in a variety of tissues [8]. It is particularly abundant in the placenta, heart, lung, pancreas and kidney [8]. With respect to nervous tissue, the channel has already been detected in a large number of regions of the central nervous system [9]. In the peripheral nervous system, it has already been shown that TRPC6 is expressed in dorsal root ganglia [10]. Here, the channel appears to play an essential pathophysiological role in pain perception [11,12,13]. To date, however, there has been no scientific work on the occurrence of TRPC6 in peripheral nerves. Although these consist of the axon processes of the sensory neurons of the dorsal root ganglia, they also contain motor nerve fibers of neurons from the spinal cord [14]. Another weakness in the data available to date is that most of the evidence for TRPC6 has been obtained from animals such as rats [11,13,15,16]. Even if human samples have indeed been analyzed in the case of the dorsal root ganglia [12], work with human tissue is much rarer and therefore only sporadic due to its difficult accessibility. In addition, the detection of TRPC6 is often based on methods such as PCR and Western blot [8,17]. Immunohistochemistry (IHC), on the other hand, offers the advantage of being able to draw conclusions about the localization of the examined antigen in the respective tissue based on the histological section [18].

The aim of this study was therefore to detect TRPC6 expression in human peripheral nerves. Immunohistochemistry was used as a method in order to be able to address possible differences in channel expression in different tissue sections.

## 2. Methods

### 2.1. Donors

The samples were taken from fixed body donors at the Anatomical Institute in Homburg (Medical Faculty of Saarland University, Homburg, Germany). Within the scope of this work, nerves were removed from a total of eight fixed specimens (Table 1). In this case, these body donors were exclusively fixed using nitrite pickling salt–ethanol–polyethylene glycol fixation.

### 2.2. Samples

In order to ensure representative sampling for the detection of TRPC6 channels in peripheral nerve tissue, both an upper limb nerve and a lower limb nerve were selected. For the upper extremity, this was the ulnar nerve and for the lower extremity, the sciatic nerve. For the ulnar nerve, the ulnar nerve sulcus located at the elbow was chosen as the harvesting site, as it is relatively easy to access anatomically. For the sciatic nerve, the decision was made to use the area proximal to the division into the tibial and fibular nerves. Samples were taken from a total of eight body donors. Both nerves were sampled on the right side from all body donors. In addition, the two nerves were sampled four times on the left side in order to obtain a lateral comparison. A total of 24 samples were thus obtained. Both longitudinal and cross-sections were prepared from each nerve, so a total of 48 histological sections were subjected to IHC staining.

### 2.3. Immunostaining

The IHC was described before in detail [19,20,21,22,23]. Briefly, after collection, the samples were fixed overnight at 4 °C in the refrigerator in a buffered 4% formaldehyde. The next day, they were transferred to phosphate-buffered saline (PBS) and stored at 4 °C for at least another 24 h. After dehydration, they were then embedded in paraffin. Sections of 7 µm thickness were then made using a microtome. Both longitudinal and cross-sections were prepared from each removed nerve. These were first stained with hematoxylin–eosin (HE) to assess the tissue quality of each series of sections before being processed for IHC. IHC started with deparaffinization and rehydration of the sections in xylene and a descending alcohol series. For antigen unmasking, the samples were then transferred to a 1% citrate buffer solution and left in a heating cabinet at 95 °C for 60 min. Next, they were cooled for 30 min at room temperature. The samples were then washed in PBS for 1 min before being incubated with normal goat serum in a humidity chamber for one hour for the blocking process. The samples were incubated with the TRPC6 antibody (Ref.: ACC017; Alomone Labs, Jerusalem, Israel) at a concentration of 1:100 and returned to the humidity chamber for 18 h at 18 °C. For each staining series, one section was selected as a negative control and incubated with rabbit serum at a dilution of 1:500 instead of the antibody. At the beginning of the second day, the samples were first washed twice in PBS for 2 min before being incubated in a humidity chamber with a 3% hydrogen peroxide solution to inactivate endogenous peroxidase for 10 min. The samples were then washed again twice in PBS for 2 min and subsequently incubated in the humidity chamber with the secondary antibody (Ref.: A10547; Invitrogen, Carlsbad, CA, USA) at a dilution of 1:500 for 60 min. After another two washes in PBS for 2 min each, staining with diaminobenzidine (DAB) (Ref.: SK-4103; Vector Laboratories, Burlingame, CA, USA) followed. The brown staining of the samples, resulting from the reaction of DAB with the horseradish peroxidase conjugated to the secondary antibody, was monitored under a microscope. Once sufficient staining was achieved, the reaction was stopped by transferring the slides into PBS. Finally, the samples were counterstained with hematoxylin before being dehydrated through an ascending ethanol series, cleared with xylene, and mounted with coverslips.

For the evaluation of the immunohistochemical staining, TRPC6 channel expression was assessed based on the brown staining of the histological sections under a light microscope. The signal intensity was categorized as strong, moderate, weak, or absent. The negative control of each staining series served as a reference. Overall, this evaluation represents a purely descriptive, semi-quantitative analysis.

## 3. Results

**Figure 1:**
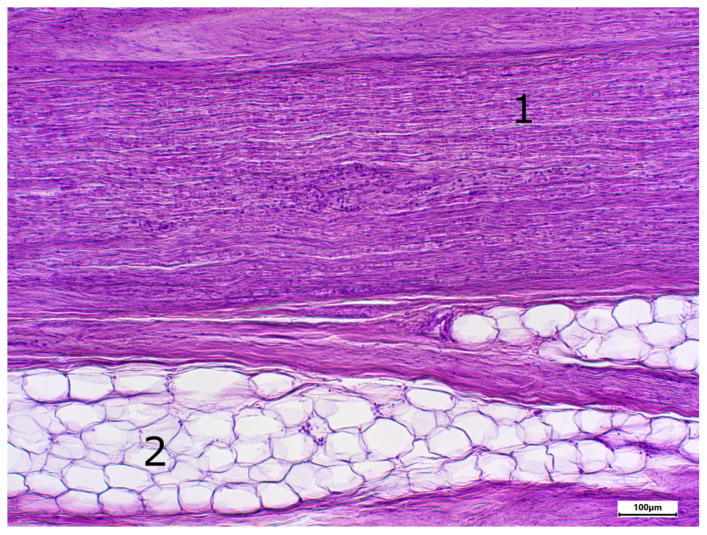
Staining with hematoxylin-eosine; body donor 1, right sciatic nerve, longitudinal section (longitudinal sectioned nerve tissue (1) and adipocytes (2)).

**Figure 2:**
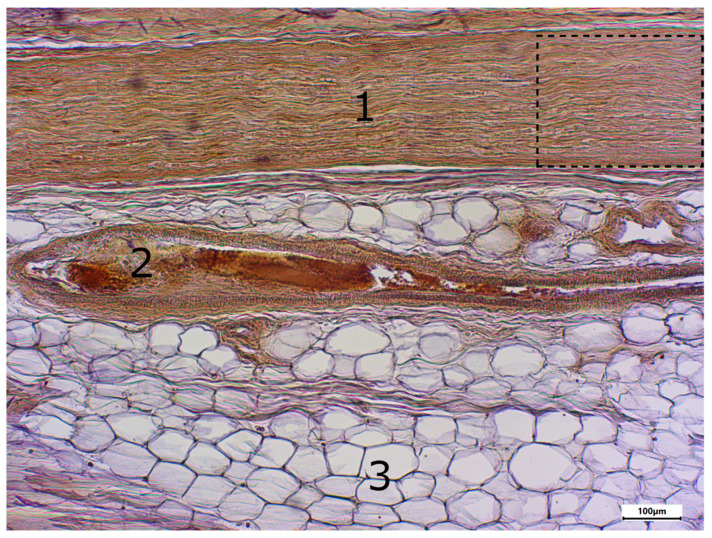
Immunostaining using the TRPC6 antibody; body donor 1, right sciatic nerve, longitudinal section (longitudinal sectioned nerve tissue (1), longitudinal sectioned blood vessel (2) and adipocytes (3)). The part in the dashed line box is presented in Figure 3.

**Figure 3:**
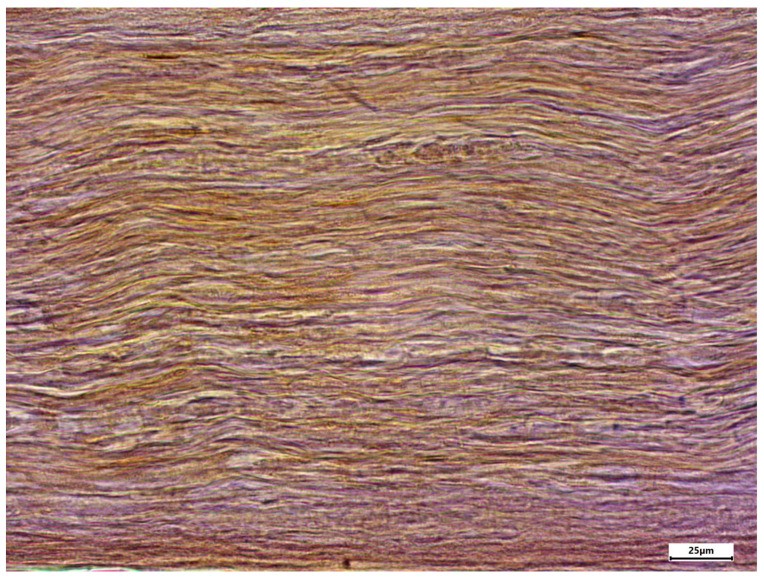
Enlarged section of Figure 2 (dashed line box); immunostaining using the TRPC6 antibody; body donor 1, right sciatic nerve, longitudinal section.

**Figure 4:**
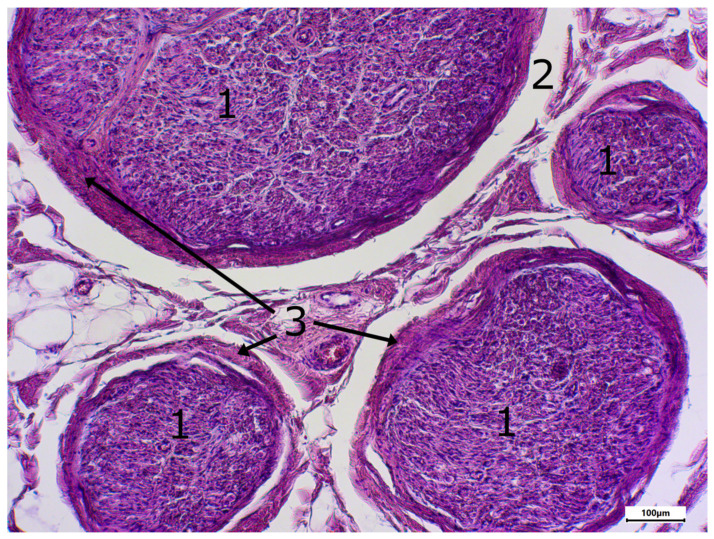
Staining with hematxylin-eosine; body donor 8, left sciatic nerve, cross-section (cross-sectioned nerve tissue (1), shrinkage gap (2), perineurium (3)).

**Figure 5:**
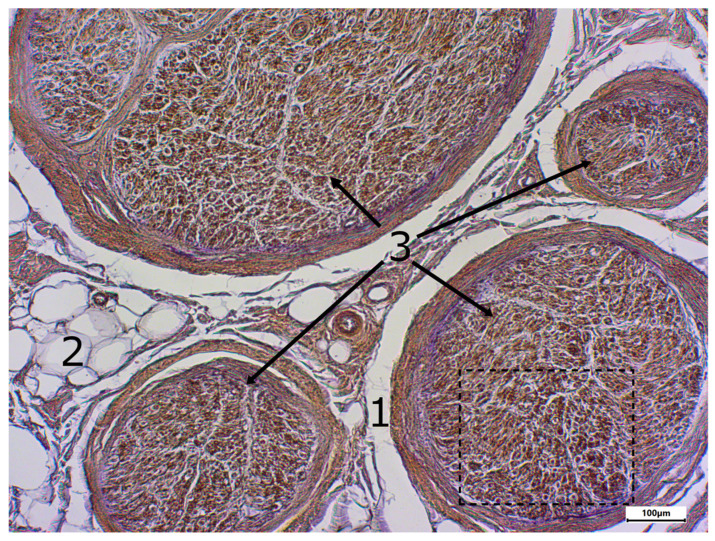
Immunostaining using the TRPC6 antibody; body donor 8, left sciatic nerve, cross-section (shrinkage gap (1), adipocytes (2) and cross-sectioned nerve tissue (3)). The part in the dashed line box is presented in Figure 6.

**Figure 6:**
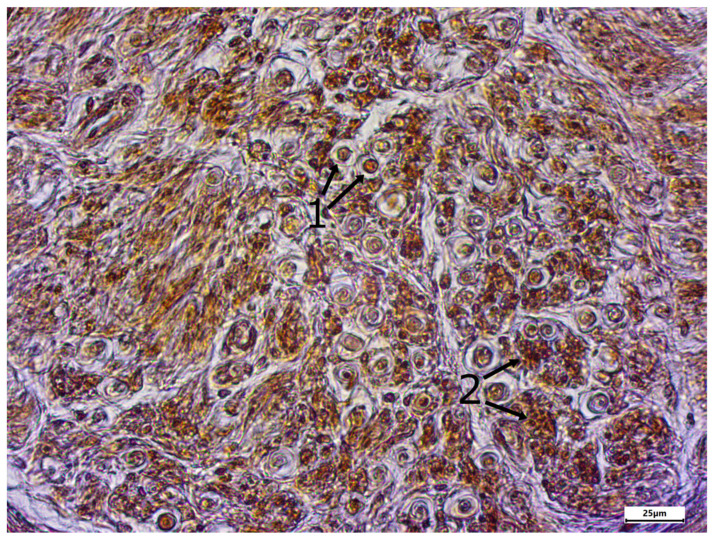
Enlarged section of Figure 5 (dashed line box); immunostaining using the TRPC6 antibody; body donor 8, left sciatic nerve, cross-section (cross-sectioned myelinated fibers (1) and cross-sectioned unmyelinated fibers (2)).

**Figure 7:**
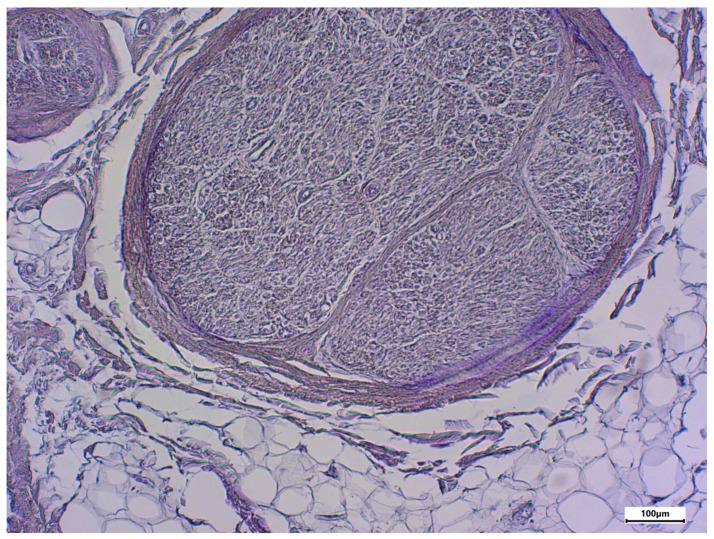
Negative control using rabbit serum; body donor 8, left sciatic nerve, cross-section.

The morphology of postmortem samples was found to be excellent (Figure 1). A total of 43 out of 48 samples showed a positive color signal (Table 2). A total of 16 sections showed a strong color signal, 16 sections showed a moderate color signal, and 11 sections showed a weak color signal. Only five sections showed no color signal (Table 2). Representative examples are presented in Figure 2, Figure 3, Figure 4, Figure 5 and Figure 6. Negative controls did not show any staining (Figure 7). Thus, the detection of TRPC6 in human peripheral nerves could be demonstrated. There were no major differences between the sciatic and ulnar nerves with regard to staining. There were also no relevant differences when comparing nerves taken from the left and right limbs. A comparison of longitudinal sections and cross-sections, on the other hand, showed that the nerve cross-sections were slightly less stained than the longitudinal sections. In the cross-sectional preparations of the ulnar nerve, there was a total of three samples, which were not stained at all in the IHC staining, whereas the longitudinal sections all displayed staining. In the sciatic nerve, there was one sample in both the cross-section and the longitudinal section that showed no signal in the IHC staining. However, there were some deviations between the different donors. For example, donors 1 and 8 showed a strong signal, whereas donors 2 and 4 exhibited weaker staining.

In addition, different areas of the histological samples were stained to different degrees. For example, the adipocytes showed no color signal, whereas the unmyelinated nerve fibers showed a strong signal. Both the perineurium and the myelinated nerve fibers showed a slightly weaker color signal.

## 4. Discussion

Overall, it can be stated that the detection of TRPC6 in the peripheral nerve tissue of the sciatic and ulnar nerves has been achieved. However, potential weaknesses in the methodology and a more nuanced evaluation of the results will be discussed in more detail below. The applied methodology is an established protocol within the research group, validated through several studies [19,20,21,22,23]. Nevertheless, possible weaknesses in the procedure will be addressed here in chronological order. The first point to mention is the varying time span between death and fixation of the body donors. During this period, decomposition processes begin, which can lead to increased protein degradation [24]. The extent of this protein degradation depends on a number of factors [24]. Important factors include the ambient temperature, age, and external conditions at the time of death [24], and, not least, the duration until final fixation [25]. As a result, the antigen may be preserved differently in various donors, leading to discrepancies in IHC staining [25]. In this study, the post-mortem interval until fixation for most body donors was between one and two days. Only for donor 2 was this interval notably longer, i.e., six days. As a matter of fact, there were two negative sections. Other samples from donor 2, however, were also positive. All body donors were fixed using NEP fixation. Therefore, differences in fixation methods do not constitute a variable in this study. Sample collection represents another potential source of error. However, the procedure was standardized in that the nerves were always collected from the same location, and the sections were consistently made along selected landmarks to ensure the highest possible precision. After collection, the samples were first stored in formalin. This leads to cross-links between the proteins and can mask their epitopes due to structural changes [26], possibly having an effect on the IHC staining. Presumably, this effect depends strongly on the duration of formalin fixation, the antibody used, and the corresponding antigen, as well as the unmasking process [27]. However, the citrate buffer used in this study was shown to have a good ability to unmask different antigens, even with different fixation times [28].

Another important aspect that has not yet been discussed is the antibody against TRPC6. As for every antibody, specific detection is also required here [29]. For this purpose, two tissue samples containing the antigen are stained using IHC [29]. One tissue sample is incubated only with the primary antibody and the second sample additionally with a control peptide, whereby the peptide corresponds to the target binding region of the antibody [29]. If the specificity of the antibody is present, the sample with additionally incubated control peptide should be significantly less stained when the two sections are finally compared [29]. As already described, the methodology of this study was a protocol that has been well established within the working group. Specificity tests for the antibody were already performed on human tissue in prior studies and, therefore, were not repeated in this series of experiments [20,21]. An even better way of demonstrating the specificity of the antibody is to use the antibody on a knockout cell line that does not express the antigen [29]. For the antibody used in this study, such a knockout validation was already performed in a study by Kistler et al. using Western blot on TRPC6-negative mice [30]. The primary antibody used in this case was a polyclonal antibody from Alomone Labs [31]. Although multiple validations were performed, the antibody still represents a deficiency in the study. Polyclonal antibodies in particular show a high variability between different batches and can therefore influence the reproducibility of the results [32]. However, even the antibody batches of monoclonal antibodies can diverge and thus also cause differences in the IHC staining [29]. Another issue in evaluating the samples was nonspecific staining. A potential cause for this could be a primary antibody concentration that was too high [33]. In this study, a concentration of the primary antibody of 1:100 was selected. Even though this concentration has already been used in some other studies [19,20,21], the optimal antibody concentration may differ from tissue to tissue [34]. To find the appropriate antibody concentration that maximizes the difference between non-specific and specific staining, different concentrations of the primary antibody can be tested using a dilution series [34]. However, such a dilution series was not carried out in this study. Due to its polyclonality, the primary antibody used is also susceptible to cross-reactions with other proteins and can therefore also contribute to increased non-specific staining [35]. The purely subjective assessment of IHC staining intensity must also be viewed critically. The system used for semi-quantitative evaluation with the differentiation of four levels (negative, weak, moderate, strong) is a widely used method [36]. However, the mere visual evaluation by inspecting the histological sections can vary due to the subjectivity of the examiner and is therefore prone to error [37]. The evaluation was further complicated by the phenomenon of color superimposition of chromogen and counterstaining with hematoxylin [38]. Finally, the section thickness of the histological preparations can also have an effect on the stainability of the samples, with thicker sections appearing to be stained more intensely [35]. To avoid this interference factor, the section thickness on the microtome was always set to 7 µm. Differential staining in the IHC may also be due to differences in channel expression [36]. Since the nerve samples were obtained from eight different donors, it can be assumed that the nerves show different levels of channel expression due to individual factors. A variety of causes are possible. Some of the possible aspects emerge from the donor data and the death certificates of the donors. Due to the small donor collective that was examined here, no definitive conclusions can be drawn about the influence that these factors have on channel expression. However, the particularly strong coloration in donor 8 was interesting. According to the death certificate, this body donor had chronic pain syndrome. Chronic pain can cause hyperalgesia and allodynia, among other things [39]. In animal experiments, the induction of such hyperalgesia was associated with increased expression of TRPC6 in dorsal root ganglia [11]. In another study, induced allodynia was also associated with increased TRPC6 expression in dorsal root ganglia [13]. TRPC6 expression was particularly increased in IB4-positive neurons [13]. They belong to the sensory neurons of fiber type C [40]. These C-fibers are unmyelinated and are, among other things, responsible for conduction [41,42]. However, conclusions on the particularly strong color signal of donor 8 cannot be drawn. Physical activity also has an influence on the expression of transmembrane proteins. Corresponding adaptation processes have been described for muscle tissue in particular [43,44,45]. However, even in nerves, exercise leads to modified expression of various channels [46]. In this regard, training has been shown to alter the transcription of genes for various receptors and ion channels in alpha motor neurons [46]. The influence of physical activity on the expression of TRPC6 in peripheral nerves is therefore quite conceivable. Diseases such as subdural hematoma can cause muscle weakness and thus lead to limited mobility [47]. According to the death certificate, immobility due to a subdural hematoma was present in donor 2, which could possibly explain the weaker color signal of the sciatic nerve in this case. However, mobility and, as a result, the activity of the musculature often decreases significantly with age [44]. In addition, a number of physiological changes occur in peripheral nerves as a result of the aging process [48]. It is also widely known that the expression of various ion channels is subject to age-related changes [49]. These points could therefore also account for differences in TRPC6 expression. In the case of this study, the color signals were somewhat weaker with increasing age, although this is only a trend. The only exception was donor 8, who suffered from chronic pain syndrome as described above.

A possible influence of gender on differences in the channel expression of TRPC6 in peripheral nerve tissue cannot be assessed in this study due to the exclusively female body donors. The relevance of TRPC6 for a variety of pathophysiological contexts is already known at the current state of research [50]. The channel also has important functions in nerve tissue [51]. In peripheral nerve tissue, in particular, it plays a key role in the development of hyperalgesia [12]. Several recent studies have already shown that increased pain perception in the form of hyperalgesia or allodynia is associated with increased expression of TRPC6 in dorsal root ganglia [11,12,13].

## 5. Conclusions

This study has demonstrated the immunohistochemical detection of TRPC6 in human samples of the ulnar and sciatic nerve whereas previous studies were limited to peripheral nerve tissue from the dorsal root ganglia and most of these samples were derived from animal tissue. So far, this is the first report on detection of TRPC6 in human peripheral nerves.

## Figures and Tables

**Table 1:** List of body donors.

Donor No.	Age	Days Until Fixation	Sex	Cause of Death
1	79	1	female	Multiple organ failure
2	89	6	female	Kidney failure
3	90	2	female	Disseminated intravascular coagulation
4	91	1	female	Aortic stenosis
5	95	2	female	Multiple organ failure
6	104	2	female	Cardiac failure
7	86	2	female	Kidney failure
8	97	2	female	Multiple organ failure

**Table 2:** Evaluation of immunostaining.

Donor No.	Ulnar Nerve				Sciatic Nerve			
	Longitudinal Section		Cross Section		Longitudinal Section		Cross Section	
	Right	Left	Right	Left	Right	Left	Right	Left
1	++		+		+++	+++	+++	+++
2	++	+++	++	−	+		−	
3	++	++	+	−	++	++	++	+
4	++		++		−		+	
5	+		++		+		+	
6	++		+		++		+	
7	+	+++	−	++	++	++	+++	+++
8	+++	+++	+++	+++	+++	+++	+++	+++

Staining intensity was categorized as strong (+++), moderate (++), weak (+) or negative (−).

## Data Availability

Stained slides are kept in archive. More microphotographs can be requested from the authors.
